# A cross-sectional survey among parents to report challenges and barriers in the administration of medicines to children in United Arab Emirates

**DOI:** 10.12688/f1000research.123317.2

**Published:** 2023-03-21

**Authors:** Nour Aliyan AlKaddour, Rawa Banoori Shah, Syed Wasif Gillani, Niloofar Hadi Sharafi, Aiman Fatima Khan, Riham Mohamed Elshafie, Hassaan Anwer Rathore

**Affiliations:** 1 Department of Pharmacy Practice, Gulf Medical University, Ajman, United Arab Emirates; 2 Clinical and Hospital Pharmacy Department, College of Pharmacy, Taibah University, Al Madinah Al Munawwarah, Saudi Arabia; 3 Clinical Pharmacy Department, ASUSH, Ain Shams University, Cairo, Egypt; 4 College of Pharmacy, QU Health, Qatar University, Doha, 2713, Qatar

**Keywords:** children, parents’ perceptions, drug administration, pediatric dosing, swallowing problems

## Abstract

**Background**: Lack of knowledge among parents can result in inappropriate administration practices.  After analyzing different studies among children, there was no data on challenges and barriers in the administration of medicines among children in this region because of the diverse environmental issues and challenges in the UAE. The objective of this study was to determine the reported administration practices of parents and challenges and barriers in the administration of medicines among children in UAE.

**Methods**: A questionnaire-based survey was conducted. A convenience sampling technique was used to collect the data. An online Raosoft® sample size calculator was applied (n = 248). The inclusion criteria were parents who had a child under 10 years of age and gave consent to participate in this study. Children with vision problems, cognitive/physical disabilities, and caregivers other than parents were excluded from this study.

**Results**: The study reported response rate of 73.2%. The mean ± S.D age of the parents in years was 35.5 ± 7.8, and the mean ± S.D of children aged years was 2.60 ± 1.54. 26.2% of parents reported treatment failure due to oral medicine administration. A total of 22.2% of parents reported that they gave medicines in doses higher than prescribed by the doctor to treat their children more quickly. Similarly, a total of 64.5% of the parents reported self-medication without consultation from a healthcare provider.

**Conclusions**: The study concluded that there were inappropriate medicine administration practices among parents. Parents reported administration of higher doses to treat their children quickly.

## Introduction

The literature shows that infectious diseases are the foremost common reason behind significant morbidity and mortality in early childhood; this is similar in developed economies where populations have high-quality housing and access to high-quality medical care. ^1^ In Africa, the top three fatalities of children under the age of five are pneumonia, diarrhea, and malaria. For children from low-income families, primary prevention of these illnesses is difficult. To reduce child deaths, accurate identification and rapid treatment with good therapy are critical. ^2^

Uneducated mothers and children delivered at home had lower healthcare utilization for diarrhea and cough. ^3^ Some long-standing issues have persisted, resulting in unacceptably high illness rates. Because of a lack of worldwide funding, feasible control methods have only lately been established. Vaccine-preventable diseases continue to be poorly controlled in many regions of the developing world. ^4^

Over-the-counter medications have become a significant issue in children. The absence of parental training, poor counseling, and lack of knowledge are the leading cause of medicine errors. Some studies also showed that the parents did not use the correct equipment to administer the medications. ^5^ According to the findings, a considerable majority of parents did not use the proper equipment to deliver medicines, utilized non-prescription pharmaceuticals, did not administer medications at the appropriate intervals, and blended medication into foods. ^6^

One of the practices in pediatrics is to mix the medicine with food. The use of bodily strength is known as forced administration. When children have a low tolerance for unpleasant tastes, open administration is used. Many parents crushed the capsules or tablets, mixed them with yogurt or meal, or dissolved them in water. ^7^

After analyzing different studies among children, there was no data on challenges and barriers in the administration of medicines among children in this region because of the diverse environmental issues and challenges in the UAE. The objective of this study is to determine the parents reported administration practices and challenges and barriers in the administration of medicines among children in the UAE.

## Methods

### Study design and setting

This study used an observation survey design to evaluate the objective. This study was conducted among general population, in addition to the outpatient department of a tertiary care hospital, in Ajman, UAE. The data was collected over seven months from October 2021 to April 2022.

### Research tool

A questionnaire-based survey was conducted to assess parents' reported challenges and barriers in administration of medicine to their children at home. It was pre-validated and adopted from the study, the consent was provided via email. ^5^

Part one of the questionnaire consisted of demographic information together with sex, residency, age, occupational status, married status, academic level, monthly financial gain, and a variety of children between six months and ten years.

Part two collected information about medications, including the following questions: who is responsible for administering medications at home, whether the child has ever refused to take tablets/pills, what to do if the child does not like taking tablets, and whether the treatment process fails because of their child do not like to take tablets, whether the treatment process failed because the child did not like to take liquid medicine and the source of information provided to the child on the medicine.

Part three consisted of information about the child, including whether the child has difficulty swallowing medicines, the types of swallowing problems, the number of times they complain about dysphagia, whether they have discussed dysphagia with the doctor, and the doctor’s recommendations.

Part four consisted of information about the practice, including questions about the tools you use to give your child prescription medicines, whether you have read the leaflet attached to the medicines, whether the dose given to the child is higher than the prescription medicine used to treat the child quickly, whether to give the child more than one, oral medicine at the same time, whether to give the child medicine without a doctor's prescription, the type of medicine used, whether the time when the child was given the medicine is recorded, when the child recovers, do the remaining medicines What's the deal. If the medicine is prescribed three times a day, they are also required to provide time for the child's medicine. All the parts of the questionnaire consist of ‘yes/no’ and multiple-response questions. ^8^

### Sample size and sampling technique

For this study's sample size, a convenience sampling technique was used to collect the data. An online Raosoft sample size calculator is applied to determine the sample size, which was 339. By assuming that the margin of error is 5%, CI 95%, a population size of 20000, and response rate of parents is 66%, according to the study conducted in UAE. ^9^

### Inclusion and exclusion criteria

The inclusion criteria were parents who had a child under ten years of age and gave consent to participate in this study. And also, parents who had children with or without acute illnesses such as bronchitis, malaria, pneumonia, diarrhea, respiratory disorders, and cough, were responsible for administering medication to their children. Children with vision problems, cognitive/physical disabilities, and caregivers other than parents were excluded from this study.

### Ethical issues

The study was approved by the Gulf Medical University Institutional Research Board (IRB) (Reference number: IRB/COP/STD/86/Oct-2021). The questionnaire content was described before giving it to the parents.

### Consent form

A written consent form was obtained from all the participants before they participated in the study.

### Primary outcomes

‐ Parents' practices during the administration of oral & liquid medications to their children at home and the acceptable behaviors of their children, how the parents overcome administration obstacles.
‐ Reported problems during the administration.
‐ Sources of parents' information.
‐ Prevalence for usage and type of non-prescribed medicines.

### Statistical analysis

The data was coded, categorized, and entered into the Statistical Package for the Social Sciences (SPSS version 21). The sociodemographic and clinical data are represented using descriptive statistics (frequency, percentage, mean, standard deviation).

## Results

The study reported a response rate of 73.2%. A total of 399 participants were invited, among them, 248 consented and completed the survey questionnaire. To treat their children more quickly, a portion of parents (22.2%) reported giving medicines in higher doses than prescribed by the doctor. When the child recovered, nearly two-thirds of the parents (46%) said they disposed of the remaining amount of medicines, while 53.6% kept it for later use.

Twenty-two percent of parents reported treatment failure due to oral drug administration. Twenty-four points two failed to administer liquid medications. A section of parents (22.2%) reported that they gave drugs in doses higher than prescribed by the doctor to treat their children more quickly. Sixty-four point five percent of parents used drugs without a prescription from a doctor. Around 47.6% of those who were interviewed reported that their children had swallowing problems during the administration of oral medications, where multiple difficulties were the most common in 40.7% of the cases.

### Sample characteristics

Table 1 shows the demographics of our research participants' parents. The mean ± SD age of parents at one was 35.5 ± 7.8, and the mean ± SD of children aged years was 2.60 ± 1.54. The majority of parents (83.9%) who completed the survey were mothers and resided in the city (97.2%). Among them, 62.9% had university-level education.

**Table 1.** Demographic information of parents (N = 248).

Characteristics	Item	Number (%)
Gender	Male	40 (16.1)
	Female	208 (83.9)
Age	<25	18 (7.3)
	25–29	40 (16.1)
	30–34	52 (21.0)
	35–39	67 (27)
	40–44	39 (15.7)
	>45	32 (12.9)
Residency	City	241 (97.2)
	Village	7 (2.8)
Participant’s educational level	Not educated	5 (2.0)
	Primary school	23 (9.3)
	Secondary school	64 (25.8)
	University	156 (62.9)
Father employment	Employed	172 (69.6)
	Unemployed	10 (4.0)
	self-employed	53 (21.5)
	Employed with medical background	12 (4.9)
Mother employment	Employed	75 (30.2)
	Non employed	144 (58.1)
	Employed with medical background	29 (11.7)
Income level of the family	<2000 AED	13 (5.2)
	2000-4999 AED	24 (9.7)
	5000-9999 AED	88 (35.5)
	>10000 AED	106 (42.7)
	NA	17 (6.9)
Health insurance	Governmental	52 (21.0)
	Private	126 (50.8)
	No insurance	70 (28.2)

NA – Not Applicable.

### Oral medicine administration at home and acceptance behaviors of children

Mothers accounted for 91.9% of those in charge of medication administration at home ( Table 2). Over half of parents surveyed said they didn't try to give their children tablets when asked about their children's acceptance behavior during oral medicine administration. When their children did not like taking tablet, 41% utilized several techniques, and 17% convinced their children to drink more water. Twenty-two percent of parents reported treatment failure due to oral medicines administration, and 14% requested another form ( Table 2).

**Table 2.** Oral medicine administration at home and acceptance behaviors of children (N = 248).

Variable	Frequency (%)
**The person responsible for medicine administration at home?**	
Father	14 (5.6)
Mother	228 (91.9)
Sister	2 (0.8)
Others *	4 (1.6)
**Did the child mind taking oral pills?**	
Yes	117 (47.2)
No	70 (28.2)
Did not try it	61 (24.6)
**What did they do when the child refused to take tablet medicines?** ^**a**^	
Drink more water	21 (17.9)
Crush capsule	3 (2.6)
Open capsule	2 (1.7)
Break capsule	1 (0.8)
Change head position	1 (0.8)
Mix with food	7 (6.0)
Mix with milk	3 (2.6)
Dissolute in water or other drinks	9 (7.7)
Request another form	18 (15.4)
Stop medicine	2 (1.7)
Give during sleep	1 (0.8)
Multiple practices	49 (41.9)
**Tablet treatment failure**	
Yes	65 (26.2)
No	67 (27)
NA	116 (46.8)
**Did the child mind taking liquid medicine?**	
Yes	109 (44.0)
No	137 (55.2)
Didn't try it	2 (0.8)
**What did they do when the child refused to take liquid medicine?** ^**b**^	
Force child	13 (11.9)
Drink more water	22 (20.2)
Mix with milk	3 (2.8)
Mix with juice	11 (10.1)
Mix with food	3 (2.8)
Stop medicine	2 (1.8)
Multiple practices	55 (50.5)
**Treatment process liquid medicine treatment failure**	
Yes	60 (24.2)
No	97 (39.1)
NA	91 (36.7)
**Source of information about medicine**	
Medical leaflet	22 (8.9)
Doctor	68 (27.4)
Nurse	1 (0.4)
Pharmacist	4 (1.6)
Old experience	1 (0.4)
Internet	6 (2.4)
Multiple sources	145 (58.5)
Others	1 (0.4)

* Health care provider.

^a^ Percentage was calculated by dividing by 117 “the number of children refused taking capsules”.

^b^ Percentage was calculated by dividing by 109 “the number of the children refused to take liquid medicines”.

NA – Not Applicable.

Forty-four percent of parents reported that children refused liquid medications, fifty-point-five percent used different practices, and eleven-point nine percent forced their children to take the liquid medicines. Twenty-four points two failed to administer the treatment.

As indicated in Table 2, the majority of parents (58.5%) got information on the medicines from numerous sources, in addition to doctors (27.3%), the Internet (2.4%), and pharmacists (1.4%).

### Swallowing problems during the administration of oral medications

Around 47.6% of those who were interviewed reported that their children had swallowing problems during the administration of oral medication, where multiple difficulties were the most common in 40.7% of the cases. Of those who reported swallowing problems, 85.6% percent discussed the problem with their doctor, who advised them to change the medicnes in most cases (32.7%) or advice to overcome the problem (30.6%) ( Table 3).

**Table 3.** Swallowing problems influencing oral medicines administration for managing childhood as reported by parents 47.6% ( *N* = 118).

Variable	Frequency (%)
**Type of problem**	
Medicines hang in the throat	18 (15.3)
Uncomfortable sense	33 (27.9)
Choking sense	11 (9.3)
Cough	8 (6.8)
Multiple difficulties	48 (40.7)
**How many times did he/she complain of that swallowing difficulty?**	
Always	54 (21.8)
Sometimes	66 (26.6)
One time	1 (0.40)
NA	127 (51.2)
**Doctor advice about the problem** ^**a**^	
Change medicines	33 (32.7)
Change dose	16 (15.8)
Give some tips to overcome the problem	31 (30.6)
Forget the problem	6 (5.9)
Multiple advice	18 (17.8)

^**a**^ Percentage calculated by dividing by 101 “the number of parents discussed swallowing problem with their doctor”.

NA – Not Applicable.

### Parents reported practices during the administration of oral medicnes

A total of 32.7% of parents used multiple tools and cups attached to administer oral liquid medicine; however, other tools were also used ( Table 4). A section of parents (22.2%) reported that they gave medicines in doses higher than prescribed by the doctor to treat their children more quickly. Almost two-thirds of the parents (46%) said that they disposed of the residual amount of the medicines when the child recovered, while 53.6% kept it for later use.

**Table 4.** Parents’ practices during the administration of oral medicines ( *N* = 247).

Variable	Frequency (%)
**A tool to give liquid medicines**	
Cup attached with medicines	81 (32.7)
Syringe	48 (19.4)
Teaspoon	25 (10.1)
Tablespoon	8 (3.2)
Other tools	4 (1.6)
Multiple tools	81 (32.7)
**Reading leaflet**	
Yes	202 (81.5)
No	45 (18.1)
**higher doses administration**	
Yes	55 (22.2)
No	192 (77.4)
**Recording time when giving the medicine**	
Yes	192 (77.4)
No	69 (27.8)

### Types of self-directed medication reported by parents

Surprisingly, sixty-four-point five percent of parents used medicine without a prescription from a doctor. Multiple medicines (104, 41.9%) are the most commonly used self-therapies, antipyretics (n:45,18.1%) ( Table 5). In the final part of the survey, the parents were asked about the interval that should be left between each dose, when a medicine is prescribed to be given three times daily, and it was revealed that 9.7% administered medication incorrectly.

**Table 5.** Types of self-therapies used by parents for their children a ( *N* =160).

Variable	Frequency (%)
Antipyretics	45 (18.1)
Antibiotics	5 (2.0)
Antiemetic	1 (0.4)
Cough medicine	5 (2.0)
Colic medicine	1 (0.4)
Influenza medicine	1 (0.4)
NA	86 (34.7)
Multiple medicine *	104 (41.9)

* Antipyretics/Antibiotics/Antidiarrheal/Laxatives/Antiemetic/Cough medicines/Colic medicines/Creams/Influenza medicine.

NA – Not Applicable.

## Discussion

This study looks at parents' practices when giving their children oral medication at home. Our research uncovered incorrect practices such as self-medication practices, using multiple medicines, higher dose administration, and inappropriate administration tools, in addition to obstacles, for example, multiple swallowing difficulties and treatment failures.

Many factors, including the disagreeable taste, can impact a child's acceptance and adherence to their prescriptions, and this can cause problems for parents when providing medications to their children. ^10^ In our study, about 47.2% of parents said their children disliked taking oral medicines, in addition to 26.2 % of tablet treatment failure. Parents try a variety of solutions to solve the problem, including mixing the medicine with milk or their children's favorite food or drink. 6 percent of parents in our survey tried mixing tablets with food, 7.7 percent dissolved tablets in water or other drinks, liquid treatment failure was 24.2, 10.1 percent tried mixing liquid medicines with juice, and 2.8 percent mixed with food and milk. When medications are used with particular foods, medicine interactions and absorption may be affected. ^11^^,^^12^

Rabia Bushra and Nousheen Aslam, conducted a review on food-medicine Interactions. After single and frequent doses of Coca-Cola, the Cmax and AUC0-alpha of ibuprofen were dramatically enhanced, indicating improved ibuprofen absorption. When taking ibuprofen with Coca-Cola, the daily dosage and frequency must be lowered. ^13^

One should note here that there might be a correlation between parents' practice in our study regarding 41.9% self-medication by parents for their children and high prevalence of self-medication with antibiotics (53%) and sedative/hypnotics (27%) was also observed among high school students in UAE. ^14^^,^^15^ Self-medication, especially non-responsible self-medication, is far from being a perfectly safe activity. Incorrect self-diagnosis, delays in obtaining medical counsel when needed, and occasional but severe adverse reactions are all potential dangers of self-medication. ^16^ Reye syndrome is a potentially fatal aspirin reaction in young infants. To avoid major adverse medication reactions, many parents are unaware that aspirin should not be administered to children under the age of 12 and should be used very carefully or not at all in adolescents aged 12–16. ^17^

Five-point one percent crush, open and break the capsules to administer them to their children. Soft gelatin capsules containing liquid should not be chewed or split since the liquid inside could be extracted, resulting in an improper dosage. Crushing medicines may lead to side effects and toxicity. ^18^

Dose errors are prevalent because dosing for children must be determined individually depending on the patient's age and weight. Non-standardized teaspoons and tablespoons lead to measurement errors. ^19^ In our study, multiple tools were used (32.7%), while 10.1% of parents used teaspoons and 3.2% used tablespoons. To reduce medication errors, the findings suggest a milliliter-only norm. ^20^^,^^21^ Acetaminophen is the most commonly prescribed pediatric analgesic and antipyretic. There are numerous accounts of significant morbidity and mortality with repeated supratherapeutic doses in the literature. ^22^ Furthermore, in our article, 22.2% of the parents gave their children medicines in higher quantities than the doctor suggested in an attempt to treat their children faster.

Counselling, medication administration instructions, and measurement tools are just a few of the areas that need to be considered, in addition to the sociodemographic characteristics of parents and children, when designing any future potential intervention aimed at reducing medication errors among children and young people at home. ^23^

The interplay of these factors - knowledge, communication, resources, and personal belief - may increase the risk of medication errors and poor health outcomes in underserved communities. These findings can be used to guide future interventions and may aid in the optimization of medication administration for pediatric patients. ^24^

Educational campaigns for responsible self-medication should be strengthened by encouraging community pharmacies to take the initiative to actively guide and educate patients about the proper use of over-the-counter medications. ^25^

Continuous efforts should be made to create age-appropriate formulations that offer both dosing flexibility and palatable taste. There is still room for improvement in terms of improving parents' health performance and increasing their knowledge of some antibiotic-related health issues. ^26^ Furthermore, because co-administration with food or liquids is still the most common method of drug administration, more detailed and explicit information about suitable vehicles should be included in the SmPC and PIL. ^27^

### Limitations

There are various drawbacks to this study. For starters, having the researcher present while answering questions may introduce uncontrollable biases. Second, because this is a cross-sectional study, causal links between variables were not possible to establish. Third, the use of convenience sampling may have skewed the results. Finally, in terms of swallowing problems, the main limitation of the existing study is the absence of data about the child's age, which is critical for distinguishing between medicine-sophisticated children and medicine-naive children. One of the limitations of our study is the difficulty in extrapolating survey findings to the entire population due to convenience sampling. There's a chance that the population is either underrepresented or overrepresented.

## Conclusions

The study concluded that the parents practice inappropriate medicine administration. It is also found that parents frequently administered higher doses to treat their children quickly. Self-medication using multiple medicines is a substantial concern.

### Recommendations

Medication errors must be made more visible to parents. It is suggested that an intervention be designed for educational programs to educate parents about medicine administration practices. When a doctor writes a prescription that must be taken multiple times per day, the intervals between doses should be indicated in hours. The primary sources of medicine information should be the doctor and pharmacist. Finally, more stringent laws make it illegal to use antibiotics without a prescription.

## Consent

Written informed consent for publication of the participants’ details was obtained from the participants.

## Data Availability

Figshare: Underlying data for ‘A cross-sectional survey among parents to report challenges and barriers in the administration of medicines to children’, https://doi.org/10.6084/m9.figshare.20208938.v1. ^8^
• Data file: Parents reported challenges and barriers in the administration of medicines among children.xlsx (Questionnaire responses) Figshare: Extended data for ‘A cross-sectional survey among parents to report challenges and barriers in the administration of medicines to children’, https://doi.org/10.6084/m9.figshare.20208938.v1. ^8^
• Supplementary file: Parents reported challenges and barriers in the administration of medicines among children.pdf (Questionnaire) Data are available under the terms of the Creative Commons Attribution 4.0 International license (CC-BY 4.0)
