# Supplementary material for: A cross-sectional survey among parents to report challenges and barriers in the administration of medicines to children in United Arab Emirates
Source: F1000Res. 2023 Mar 21;11:1431. Originally published 2022 Dec 5. [Version 2] doi: 10.12688/f1000research.123317.2 (PMC10090859; doi:10.12688/f1000research.123317.2)
Supplement: Peer review history

# Peer review: Reviewer response for version 2

**DOI:** 10.5256/f1000research.144932.r167159

**Published:** 2023-04-11

**Authors:** Subish Palaian

**Affiliations:** 1 Department of Clinical Sciences, College of Pharmacy and Health Sciences, Ajman University, Ajman, United Arab Emirates

Authors performed the revision

Is the work clearly and accurately presented and does it cite the current literature?

Partly

If applicable, is the statistical analysis and its interpretation appropriate?

Partly

Are all the source data underlying the results available to ensure full reproducibility?

Yes

Is the study design appropriate and is the work technically sound?

Yes

Are the conclusions drawn adequately supported by the results?

Partly

Are sufficient details of methods and analysis provided to allow replication by others?

Yes

Reviewer Expertise:

Rational use of medicines

I confirm that I have read this submission and believe that I have an appropriate level of expertise to confirm that it is of an acceptable scientific standard.

# Peer review: Reviewer response for version 1

**DOI:** 10.5256/f1000research.135412.r157320

**Published:** 2023-02-01

**Authors:** Amjad Khan

**Affiliations:** 1 Department of Pharmacy, Quaid-i-Azam University, Islamabad, Pakistan

Thank you for the invitation to review this study. The authors made a good effort to explore the parents' reported challenges on drug administration among children in UAE. I have a few minor comments on this manuscript.

1. Abstract requires revisions on highlighted the important findings only. 
2. Whole manuscript requires copy and language editing for minor grammatical and syntax errors.
3. Provide limitations of the study specifically related to connivence sampling technique 
4. Each table and graph should have a highlighted summary in the beginning. Also provide abbreviations in the end of each table. 
5. Results section need minor revision with sequence of data according to objectives of the study. 
6. Please add recent references in the discussion to support the findings.

Is the work clearly and accurately presented and does it cite the current literature?

Yes

If applicable, is the statistical analysis and its interpretation appropriate?

Yes

Are all the source data underlying the results available to ensure full reproducibility?

Yes

Is the study design appropriate and is the work technically sound?

Yes

Are the conclusions drawn adequately supported by the results?

Yes

Are sufficient details of methods and analysis provided to allow replication by others?

Yes

Reviewer Expertise:

Clinical Pharmacy, Public Health, Clinical Trials

I confirm that I have read this submission and believe that I have an appropriate level of expertise to confirm that it is of an acceptable scientific standard.

# Response

**Published:** 2023-02-27

**Authors:** Syed Wasif Gillani

**Affiliations:** Gulf Medical University, United Arab Emirates

Response to the reviewer:

1. Abstract changes with only highlighted outcomes.
2. The manuscript's language and grammar are revised and edited.
3. Three study limitations, particularly those related to the concordance sampling technique, were added.
4. A summary is already provided, and abbreviations were added.
5. The Results section is now written based on the objectives of the study.
6. updated references were added.

# Peer review: Reviewer response for version 1

**DOI:** 10.5256/f1000research.135412.r157323

**Published:** 2023-01-13

**Authors:** Subish Palaian

**Affiliations:** 1 Department of Clinical Sciences, College of Pharmacy and Health Sciences, Ajman University, Ajman, United Arab Emirates

I take this opportunity to thank the Editor for giving me an opportunity to review this manuscript. It is novel research. There is a scope for improvement in the manuscript. Here are my comments:

**Title:**

 The title should mention ‘United Arab Emirates'

**Abstract:**

 The first line is not properly written. It may be edited as …. inappropriate medicine administration practices. I suggest the authors use the term ‘medicine’ instead of ‘drug’. The results should focus on more significant findings.

**Introduction:**

 This section should clearly specify the reasons for undertaking the study. The study rationale is not clear. The authors are expected to provide more literature evidence to justify the study rationale. A more detailed literature review covering UAE and neighboring countries is needed. There is a scope for adding more information in this section. All possible similar studies should be cited.

**Methods:**

 Study design, This study will be conducted……. The term ‘will be’ has to be edited. Further, ‘public places’ is not relevant. Authors can remove it.

**Research tool:**

 More detail information on the preparation, validation, and reliability analysis of the tool is needed. If the tool used is an adopted one, hence permission for the use of the tool and references have to be provided.  

**Statistical analysis:**

 The version number of SPSS has to be provided. There is a scope for performing more inferential statistical analysis to arrive at more concrete findings based on the p values.

**Discussion:**

 This section is too short. More literature evidence should be compared with the current findings. The discussion should also focus on the study findings and its significances for future practices. Any potential policy implications based on the study findings have to be explored. Since the study is conducted in the UAE, the discussion must focus on literature and practices from UAE.

**Limitations:**

 A convenient sampling method followed in the research must be added as a limitation.

**References:**

 Authors are required to provide more recent references wherever possible.

Is the work clearly and accurately presented and does it cite the current literature?

Partly

If applicable, is the statistical analysis and its interpretation appropriate?

Partly

Are all the source data underlying the results available to ensure full reproducibility?

Yes

Is the study design appropriate and is the work technically sound?

Yes

Are the conclusions drawn adequately supported by the results?

Partly

Are sufficient details of methods and analysis provided to allow replication by others?

Yes

Reviewer Expertise:

Rational use of medicines

I confirm that I have read this submission and believe that I have an appropriate level of expertise to confirm that it is of an acceptable scientific standard, however I have significant reservations, as outlined above.

# Response

**Published:** 2023-02-27

**Authors:** Syed Wasif Gillani

**Affiliations:** Gulf Medical University, United Arab Emirates

Response to reviewer

1.  Tittle is changed.
2. Abstract is updated.
3. New points were added for the introduction, in addition to references.
4. Proper grammar in the study design.
5. Research tool section now explains the permission
6. Spss version is updated, in this study we are focused on descriptive analysis, but we are planning to conduct data from different parts in UAE and inferential statistics.
7. Discussion references are added from neighbouring countries.
8. Limitations: Convenient sampling method was added as a limitation.
